# As Others See Us

**Published:** 1920-04-10

**Authors:** 


### As Others See Us.

The modern nurse is hard and jolly,
Deems gentle womanliness folly:
As easy find the Lady of the Lamp
In Sister Betsy Prig or Matron Gamp.

We print this squib by the Rev. S. Claude Tickell because, whether intentional or not, there is a shrewd criticism in its satire. The modern young woman, and therefore the modern nurse, often acts on the assumption that if she is only jolly her character will take care of itself. It is well for her therefore to be reminded that a woman who is merely jolly is almost invariably a hard woman; and a hard woman is the best definition of a bad nurse

